# Ginseng (*Panax quinquefolius*) and Licorice (*Glycyrrhiza uralensis*) Root Extract Combinations Increase Hepatocarcinoma Cell (Hep-G2) Viability

**DOI:** 10.1093/ecam/nep074

**Published:** 2011-05-02

**Authors:** David G. Popovich, Shi Yun Yeo, Wei Zhang

**Affiliations:** Department of Chemistry, National University of Singapore, Science Drive 4, Singapore 117543

## Abstract

The combined cytoactive effects of American ginseng (*Panax quinquefolius*) and licorice (*Glycyrrhiza uralensis*) root extracts were investigated in a hepatocarcinoma cell line (Hep-G2). An isobolographic analysis was utilized to express the possibility of synergistic, additive or antagonistic interaction between the two extracts. Both ginseng and licorice roots are widely utilized in traditional Chinese medicine preparations to treat a variety of ailments. However, the effect of the herbs in combination is currently unknown in cultured Hep-G2 cells. Ginseng (GE) and licorice (LE) extracts were both able to reduce cell viability. The LC50 values, after 72 h, were found to be 0.64 ± 0.02 mg/mL (GE) and 0.53 ± 0.02 mg/mL (LE). An isobologram was plotted, which included five theoretical LC50s calculated, based on the fixed fraction method of combination ginseng to licorice extracts to establish a line of additivity. All combinations of GE to LE (1/5, 1/3, 1/2, 2/3, 4/5) produced an effect on Hep-G2 cell viability but they were all found to be antagonistic. The LC50 of fractions 1/3, 1/2, 2/3 were 23%, 21% and 18% above the theoretical LC50. Lactate dehydrogenase release indicated that as the proportion of GE to LE increased beyond 50%, the influence on membrane permeability increased. Cell-cycle analysis showed a slight but significant arrest at the G1 phase of cell cycle for LE. Both GE and LE reduced Hep-G2 viability independently; however, the combinations of both extracts were found to have an antagonistic effect on cell viability and increased cultured Hep-G2 survival.

## 1. Introduction

American ginseng (*Panax quinquefolius*) and Asian licorice (*Glycyrrhiza uralensis*) roots have a long history of use in traditional Chinese medicine (TCM). These roots are often used in combination with other herbal ingredients with the goal of improving the effectiveness of the TCM preparations. Ginseng and licorice roots are likely the most utilized TCM ingredients [1], and both roots are also now utilized by the nutraceutical and functional food industries for their purported bioactive and functional properties [2–4].

The main bioactive ingredients found in ginseng are thought to be a group of dammarane triterpene saponins, which are also known as ginsenosides [5], and one of the main active ingredient in licorice is glycyrrhizic acid, which is an oleanane triterpene saponin. Both roots also contain a variety of other phytochemical compounds such as polysaccharides and polyphenols [4, 6] that may add additional benefits to tinctures or decoctions.

Licorice root extracts and glycyrrhizic acid have been reported to have both cytotoxic and hepato-protective properties. Glycyrrhizic acid extracted from licorice root was reported to protect against aflatoxin B_1_ injury in Hep-G2 cells [7] and reduced free fatty acid-induced hepatic lipotoxicity in both cultured Hep-G2 cells and high-fat-diet-induced lipotoxicity in rats [8]. Furthermore, licorice and glycyrrhizic acid protected primary hepatocytes against azathioprine (1 *μ*M) injury, an immune suppressant drug that has hepatic side effects [9]. Glycyrrhizic acid has also been clinically used in Japan to treat chronic hepatitis [10]. Licorice extracts have exhibited anti-inflammatory activity and inhibited cultured hepatic carcinoma cell (Hep-3B) proliferation [3]. A licorice extract also reduced prostate specific antigen release, and reduced cultured prostate cancer cell line (LNCaP) proliferation [11] and is one of seven ingredients in the herbal supplement PC-SPES [12].

Ginseng root and ginsenosides have been associated with a wide range of biological activities such as anti-diabetic [13, 14], immune stimulation [15, 16] and an ability to inhibit the growth of variety of cultured cancer cells [5, 17, 18]. The precise mechanism attributed to ginseng and ginsenoside cellular cytotoxicity is not entirely clear but an interaction with the cell membranes leading to membrane permeation has been previously reported [18].

The objective of this study was to determine if the combination of American ginseng (*Panax quinquefolius*) and licorice extracts (*Glycyrrhiza uralensis*) would synergistically or antagonistically affect the viability of cultured hepatocarcinoma cell line (Hep-G2). An isobolographic analysis was utilized to efficiently depict synergistic, antagonistic and additive responses in Hep-G2 cells. Isobolographic analysis provides a clear graphical view of the interactions between the two components and has been utilized to depict interactions between two drug combinations [19].

## 2. Methods

### 2.1. Plant Materials

Dried ginseng (*Panax quinquefolius*) and licorice (*Glycyrrhiza uralensis*) roots were purchased locally (Singapore). Ginsenoside HPLC standards (Rg1, Re, Rb1, Rc, Rd) were purchased from Chromadex Inc. (Santa Ana, CA, USA) and glycyrrhizic acid standard was purchased from Sigma (Steinheim, Germany), trifluoroacetic acid (TFA) was obtained from Merck (Whitehouse Station, NJ, USA). Acetonitrile and methanol were of HPLC grade (Tedia Inc., Fairfield, OH, USA), ethanol was of analytical grade (Fisher Scientific, UK).

Dried ginseng root (*Panax quinquefolius*) was ground into a powder and refluxed in methanol (500 mL) for 3 h, filtered twice (Whatman no 4) and the extraction was repeated three times. The solution was concentrated under vacuum, and the extract was applied to a preconditioned polymeric absorbent Amberlite XAD-4 (Sigma, St. Louis, MO, USA) column (pore diameter of 40 Å, bed volume of 60 cm^3^, flow rate of 10 mL/min) and washed with distilled water (1 L) to remove polar compounds as previously described [20]. Ginsenosides were eluted from the column using absolute ethanol (500 mL) and concentrated under vacuum, lyophilized and is herein referred to as the ginseng extract (GE).

Powdered dried licorice (*Glycyrrhiza uralensis*) root was refluxed for 2.5 h in methanol (70%, 400 mL), filtered twice, extracted three times and concentrated under vacuum and is herein referred to as the licorice extract (LE).

### 2.2. HPLC Analysis

A Waters HPLC (Milford, MA, USA) equipped with quaternary gradient pump and a photodiode-array (PDA) detector was used to assess the amount of either ginsenosides or glycyrrhizic acid in the respective extracts. The HPLC analysis was conducted using a reverse-phase C18 column (C18, 4.6 × 250 mm, 5 *μ*m particle size, Waters) with a column temperature of 25°C, injection volume of 20 *μ*L and detection wavelength of 203 nm for ginsenosides and 254 nm for GA. GE, ginsenosides standards, LE and GA were separately dissolved in methanol, filtered through a 0.45 *μ*m syringe filter (Minisart, Germany), prior to analysis. HPLC analysis for GE consisted of two separate gradient programs (referred to as gradients 1 and 2), due to the similar retention times of ginsenosides Rg1 and Re. Both solvent programs consisted of the combination of water (A) and acetonitrile (B). Gradient program 1 (percentage acetonitrile and flow rate are enclosed in parenthesis) was as listed: Time 0 (20% B, 1 mL/min), 20 min (25% B, 0.7 mL/min), 40 min (49% B, 0.7 mL/min), 50 min (100% B, 0.7 mL/min), and 60 min (80% B, 0.7 mL/min). Gradient program 2 consisted of: Time 0 (20% B, 1 mL/min), 45 min 22% B, 0.7 mL/min), 50 min, (60% B, 0.7 mL/min), 60 min (20% B, 0.7 mL/min).

HPLC separation gradient program for LE and GA utilized 0.5% TFA water (A) and acetonitrile (B) and was as follows: Time 0 (20% B), 5 min (40% B), 10–20 min (50% B), and the flow rate was constant at 0.8 mL/min. Ginsenoside Rg1, Re, Rb1, Rc, Rd in the GE and GA in the LE were identified according to the respective standard curves and were assessed in triplicate.

### 2.3. Cell Culture

Human hepatocarcinoma (Hep-G2) cells were purchased from American Type Culture Collection (Manassas, VA, USA). The cells were maintained in Dulbecco's modified Eagle's medium (DMEM) supplemented with 10% fetal bovine serum (Sigma), 100 U penicillin, and 100 *μ*g/mL streptomycin (Gibco, Invitrogen, Canada) in a humidified atmosphere of 5% CO_2_ at 37°C. Cells were maintained at a concentration between 2 × 10^5^ and 1 × 10^6^ cells/mL. Cells were subcultured every 2-3 days by total medium replacement using 0.25% (w/v) trypsin −0.53 mM EDTA solution (GIBCO). Viable cells were assessed by 0.04% trypan blue exclusion dye (MP Biomedicals, Solon, OH, USA) using a hemocytometer and assessed in quadruplicate.

### 2.4. Cell Viability MTT Assay

Cell viability was measured by 3-(4,5-dimethylthiazol-2-yl)-2,5-diphenyl tetrazolium bromide, MTT (Sigma) assay, in order to establish an LC50 value (concentration to inhibit 50% of cells). Cells were seeded at a concentration of 1 × 10^5^ cells/well in a 96-microwell plate and were allowed to adhere for 24 h. Stock solutions of GE and LE (1.0 mg/mL) were dissolved and filtered through a sterile 0.2 *μ*m syringe filter (Millex GP, Ireland) before adding to the cells. Extracts were added at various concentrations (0.2–0.9 mg/mL) and incubated for 72 h. Controls contained Hep-G2 cells, culture medium, but no extracts. At the end of 72 h, the extract-containing medium was removed and 100 *μ*L of 0.5 mg/mL of MTT was added and incubated for 4 h as previously described [21], crystals were solubilized in 100 *μ*L of 10% SDS (National University of Medical Institution, Singapore) in 0.01 N HCl and incubated overnight. The absorbance was measured at 550 nm using a microplate reader (Multiskan Spectrum, Thermo Electron Corporation, Waltham, MA, USA) and the results were expressed as the percentage of viable cells with respect to the untreated control cells. Cell viability (%) was calculated as (mean absorbance of the sample/mean absorbance of the control) × 100.

### 2.5. Isobologram Analysis

The interactions between GE and LE were analyzed with the use of an isobologram and test of significance according to the method of Tallarida (2000) [22]. The LC50 values for ginseng and licorice extracts described above were used to plot the isobologram. The LC50 value for GE was plotted on the *x*-axis, while the LC50 value for LE on the *y*-axis. The line connecting the two points is referred to as the line of additivity and represents the predicted LC50 (refer to (1)) of different combinations of extracts. Antagonistic combinations are points that lie above the line, while synergistic combinations are points below the line [22]. The fixed ratio design was used. Briefly, five fractions (1/5, 1/3, 1/2, 2/3, 4/5) of GE to LE were used to test the interactions of the two extracts (refer to (2)). LC50 values were first obtained for GE and LE alone and then the LC50 values were obtained for each of the five fractions.

### 2.6. Cell LDH Activity

Hep-G2 cells were seeded at a concentration of 5 × 10^5^ cells/mL in 24-well plates and allowed to adhere for 24 h. The medium was removed and extracts (ginseng, licorice or the five fractions) at their respective LC50 concentration were added to the wells. The concentrations used were 0.64 mg/mL (GE), 0.53 mg/mL (LE), 0.64 mg/mL (f1/5), 0.70 mg/mL (f1/3), 0.74 mg/mL (f1/2), 0.74 mg/mL (f2/3) and 0.69 mg/mL (f4/5). Cells were incubated for 24, 48 and 72 h, and untreated cells acted as control. At the end of each incubation time, the medium was removed and tested for LDH activity as previously described [5].

### 2.7. Cell-Cycle Analysis

GE, LE and five fractions (1/5, 1/3, 1/2, 2/3, 4/5) were added to Hep-G2 cells at their respective LC50 concentrations. Cells were incubated for 24, 48 and 72 h with untreated cells acting as controls. Cell-cycle analysis was determined as previously described [21]. Briefly, cells were trypsinized, washed with PBS and the cellular pellet was fixed in ice-cold 70% ethanol overnight at 4°C. The supernatant was removed by centrifugation, 1 mL of PBS containing 50 *μ*g/mL propidium iodide (Sigma) and 100 U/mL RNAse A (Applichem Inc, Cheshire, CT, USA) were added and incubated at room temperature for 1 h before data acquisition using Dako Cytomation Cyan LX Flow Cytometry (Beckman Coulter, Fullerton, CA, USA) and analyzed with Dako Summit v4.3 software package.

### 2.8. Statistical and Data Analysis

The LC50 values of GE, LE and the five fractions were determined from five separate experiments with five replicates for each experiment. LDH and cell-cycle analysis were repeated on three separate occasions with three replicates. Data are expressed as mean ± standard deviation (SD). A one-way ANOVA with Tukey post hoc comparison of means was used to test for significance (*P* < .05) of LDH and cell-cycle analysis using the SPSS statistical software (v12.0, Chicago, IL, USA).

**Determination of Theoretical LC50 (CAL50)**

(1) $$\begin{array}{c} \text{CAL}50=f\text{GE}+\left(1-f\right)\text{LE} \end{array}$$

GE and LE variables represent the experimentally determined LC50 values of ginseng and licorice extracts, and variable *f* represents the fix ratio fractions (1/5, 1/3, 1/2, 2/3, 4/5) of GE to LE combinations, CAL50 is the theoretical or calculated LC50.

**Determination of Concentrations of Individual Extract in Each Fraction**

(2) $$\begin{array}{c} {\text{GE}}_{f}=\frac{f\text{GE}}{\text{CAL}50},\\ {\text{LE}}_{f}=\frac{\left(1-f\right)\text{LE}}{\text{CAL}50} \end{array}$$

Variables *f*, GE, LE and CAL50 are described above, GE*_f_* and LE*_f_* represent the amount each root extract to add to each fraction.

**Additive, Synergy and Antagonism**

(3) $$\begin{array}{c} \text{Additive:}\;\;\text{CAL}50-\text{EXPLC}50=0\\ \text{Synergy:}\;\;\text{EXPLC}50<\text{CAL}50\\ \text{Antagonism:}\;\;\text{EXPLC}50>\text{CAL}50 \end{array}$$

Variable EXPLC50 is the experimentally derived LC50 of the fractions and variable CAL50 is described above. The test of significance and the above equations are based on the reported literature [22, 23].

## 3. Results

### 3.1. HPLC Analysis

Individual ginsenoside analysis showed that the percentage of ginsenosides in the GE was determined to be Re (20.2 ± 7.8), Rb1 (8.8 ± 0.2), Rg1 (1.5 ± 0.04) and Rd (0.71 ± 0.01), and the total ginsenosides content was determined to be 30.9 ± 7.8% (dry weight). Ginsenoside Rc was detected but the amount was below the limit of detection. For LE, the total percentage of glycyrrhizic acid was determined to be 7.1 ± 0.2% (dry weight).

### 3.2. Dose-Response LC50 Determination of Ginseng and Licorice Extracts

**Figure 1:**
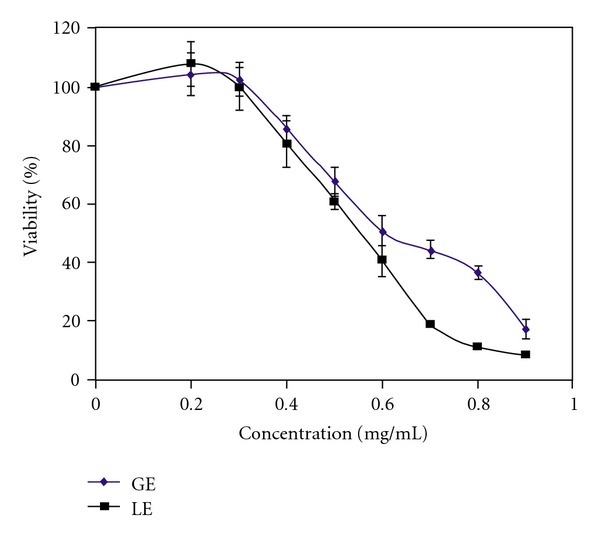
The effect of GE and LE on Hep-G2 viability. The results are expressed as mean ± SD of five separate experiments with five replicates.

Figure 1 shows the dose-response relationship of GE and LE. The LC50 was calculated by plotting cell viability (%) versus log concentration (graph not shown) which yielded a linear equation of *y* = −164.32*x* + 511.58 (*r* ^2^ = 0.982) for GE and *y* = −218.84*x* + 646.88 (*r* ^2^ = 0.981) for the LE. The LC50s were determined to be 0.64 ± 0.02 mg/mL for GE and 0.53 ± 0.02 mg/mL for LE. Both GE and LE showed a dose dependent effect on Hep-G2 viability, and the LE LC50 value was found to be significantly (*P* < .05) lower LC50 compared to GE.

### 3.3. Dose-Response LC50 Determination of Fractions

**Figure 2:**
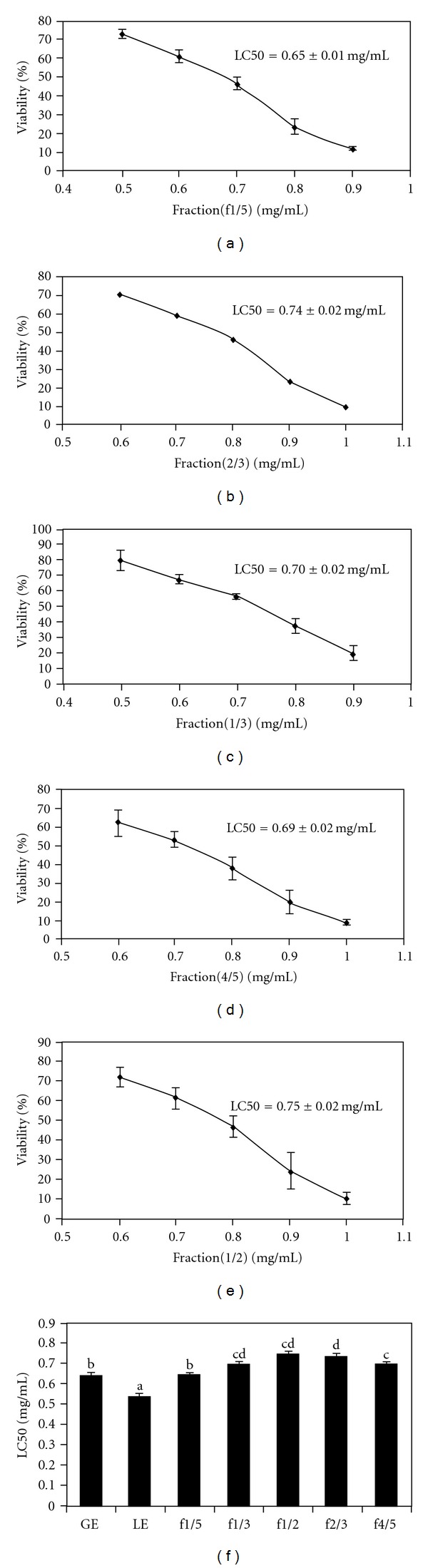
The effect of five distinct fractions (1/5, 1/3, 1/2, 2/3, 4/5) on Hep-G2 viability (a)–(e). (f) corresponds to the LC50s values of GE, LE and five fractions. Bars with different letters are significantly different from each other (*P* < .05). Results are expressed as mean ± SD of five separate experiments with five replicates.

The LC50 values of five fractions corresponding to a ratio of ginseng to licorice extracts of 1/5, 1/3, 1/2, 2/3 and 4/5 and are shown in Figures 2(a)–2(e)) and calculated as described above. Compared to the LC50 of GE, fractions (1/3, 1/2, 2/3, 4/5) were all significantly (*P* < .05) greater than GE with the exception of f1/5 which was similar to GE. Furthermore, the LC50 for all fractions were significantly (*P* < .05) greater when compared to the LC50 of LE (Figure 2(f)).

### 3.4. Isobolographic Analysis

**Figure 3:**
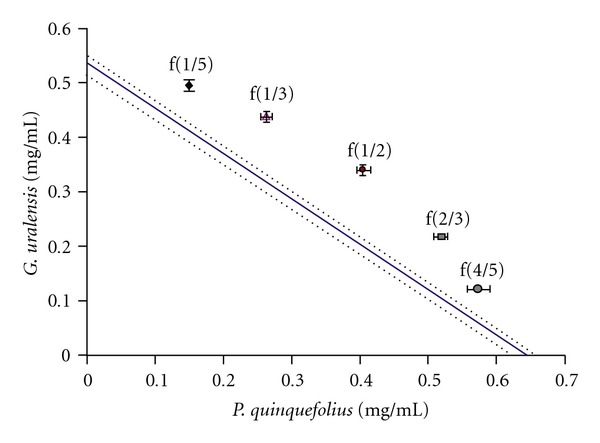
Isobolographic representation of the interaction between experimentally measured GE, LE and fractions (graphical points) and the theoretical line of additively (solid line) on Hep-G2 viability. The dotted line represents the theoretical standard deviation of plus/minus 2.5%. Experimental points and error bars are expressed as mean ± SD of five separate experiments with five replicates.

Figure 3 represents a graphical view of the effects of the combinations of GE and LE on Hep-G2 cells viability. An isobologram was created using the LC50 value for GE, which intersects the *x*-axis, and the LC50 value for LE, which intersects the *y*-axis. The two points are joined together to form the line of additivity. This line of additivity refers to the concentrations of GE and LE extracts needed to produce the same effect if they were to act alone. Points lying on the line are termed additive, above the line are antagonistic and below the line are synergistic [22, 23]. All combinations (1/5, 1/3, 1/2, 2/3 and 4/5) of GE and LE showed antagonism. They were all greater than the line of additivity. Fractions 1/2 and 2/3 showed the greatest antagonistic effect.  Table 1 shows the theoretical LC50 and the experimentally observed LC50. Fractions 1/3 and 1/2 showed the greatest difference from the theoretical LC50 at 23 and 21%, respectively.

### 3.5. Cell-Cycle Analysis

**Figure 4:**
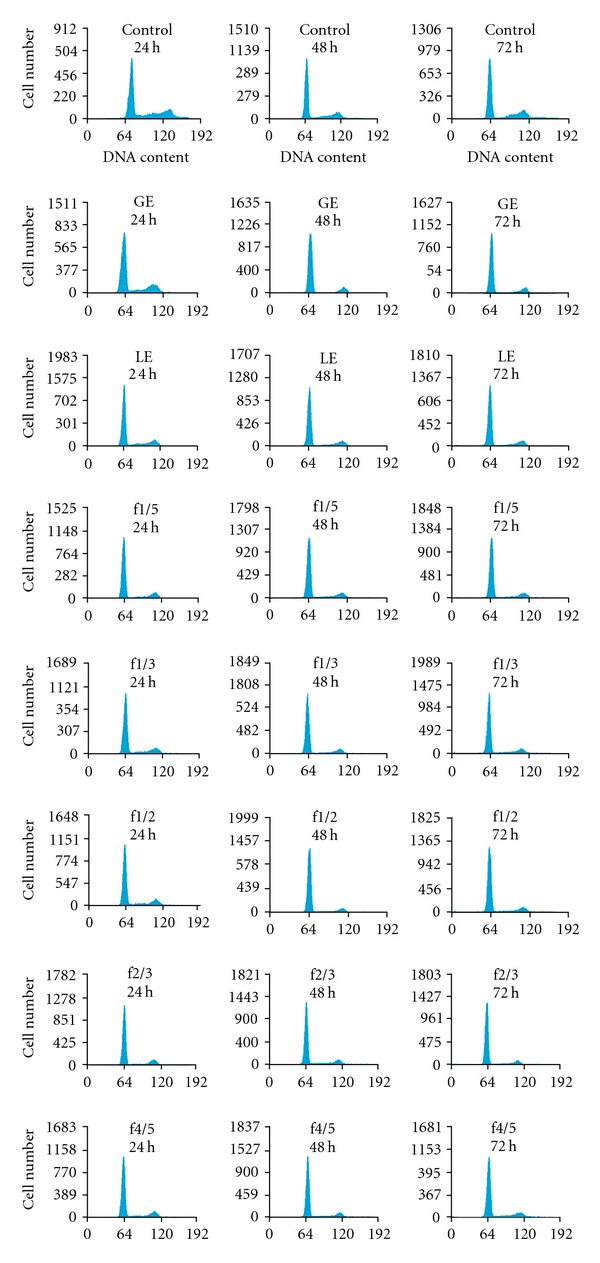
DNA cell-cycle histograms of Hep-G2 cells. Cells were treated with GE, LE and five fractions (1/5, 1/3, 1/2, 2/3, 4/5) for 24, 48 and 72 h, respectively, at the respective LC50s. Untreated cells acted as controls. Cells were fixed in 70% ethanol and stained with PI as described in the Methods section. DNA histograms shown are representatives of the assay repeated in three independent experiments with similar results.

Representative DNA histograms of the various treatments are shown in Figure 4 and corresponding analysis is listed in Table 2. Apoptotic cells (sub G1) were generally not observed during the cell-cycle analysis. A majority of fractions tested did not show any significant increase of apoptotic cells at 24 h of treatment as compared to the untreated control. GE significantly (*P* < .05) increased sub G1 cells but the increase was only marginal at 48 and 72 h. Overall, Hep-G2 cells generally showed a significant (*P* < .05) increase in the proportion of cells in the G1 phase at 24 and 48 h time periods with the exception of GE at 24 h. A decrease in the proportion of G2/M cells was observed for all extracts and combinations compared to the control. At 48 and 72 h of treatment, significant reductions (*P* < .05) in the proportion of G2/M cells were observed in fractions 1/3, 1/2, 2/3 and 4/5. Cells observed in the S phase were reduced but not significant with the exception of fractions 1/3, 1/2, 2/3 and 4/5 after 24 h of treatment.

### 3.6. LDH Analysis

**Figure 5:**
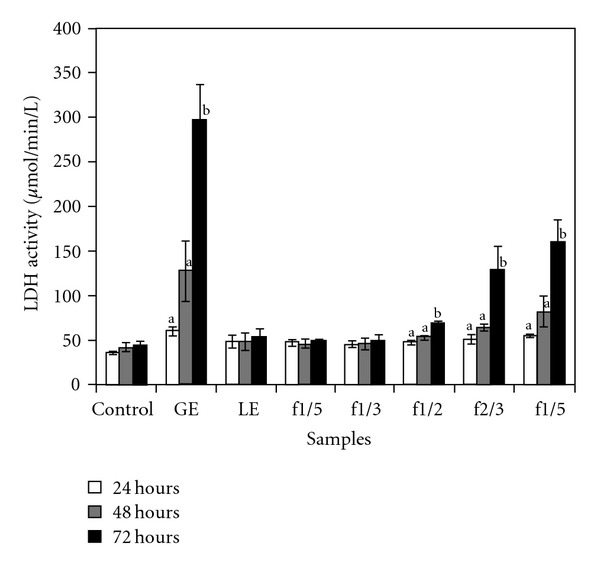
The effect of GE, LE and five fractions (1/5, 1/3, 1/2, 2/3, 4/5) on LDH activity. Cells were treated with different extracts for 24, 48 and 72 h at their respective LC50 concentration determined from a 72-h MTT assay. Results are expressed as mean ± SD with three replicates repeated in three separate experiments. Untreated cells acted as controls. Bars with different letters are significantly different (*P* < .05).

The effect of different treatments and exposure times on LDH release, a marker of membrane integrity and damage, is shown in Figure 5. LE treatment from 24 to 72 h did not significantly affect the release of LDH compared to the corresponding control cells. GE treatment produced the greatest significant increase in LDH release, while fractions that contained a proportion of at least one half GE (e.g., f1/2, f2/3, f4/5) had significant (*P* < .05) increase in LDH release compared to untreated control cells after 72 h of incubation. Fractions 1/2, 2/3 and 4/5 were found to have a percentage increase of 57% (f1/2), 192% (f2/3) and 263% (f4/5). GE showed greatest significant LDH increase at all time periods followed by f4/5 at 48 h and fractions f2/3 and f4/5.

## 4. Discussion

By utilizing an isobolographic analysis, we have effectively shown that the combinations of ginseng (*Panax quinquefolius*) and licorice (*Glycyrrhiza uralensis*) extracts do not produce a synergistic effect on reducing Hep-G2 cell viability. On the contrary, the effect was antagonistic. Fractions 1/2 and 2/3 were found to have the highest LC50 values (0.75 ± 0.02 and 0.74 ± 0.02 mg/mL, resp.), compared to the other fractions and fractions 1/3 and 1/2 showed the greatest percentage difference when compared to the theoretical or calculated LC50 (CAL50). Adams et al. [19] also reported antagonism with the combination *G. uralensis* with either *Rabdosia rubescens* or *Scuteellaria baicalensis* in equal parts (1 : 1 fraction) in cultured prostate cancer cells. *Glycyrrhiza uralensis, R. rubescens* and *S. baicalensis* are also found in TCM prescriptions [24] and PC-SPES formulations [12]. *Rabdosia rubescens* and *S. baicalensis* have been reported to reduce cultured cancer cell viability in a number of cell lines (Hep-G2, MCF-7, MCF-10A) [25, 26].

In this study, ginseng extract increased the release of LDH at the LC50 concentration over time with a significant (*P* < .05) increase at 48 and 72 h of exposure, whereas licorice extract did not. Ginseng extract alone has been shown to be effective in permeating the cellular membrane of intestinal cells (Int-407, caco-2 cells) [18] thereby releasing LDH from the cytoplasm possibly through an interaction with membrane cholesterol [27]. Notably, when the proportions of ginseng to licorice contained at least 50% ginseng (f1/2, f2/3, f4/5) a significant (*P* < .05) increase in LDH release was observed at 72 h for ginseng and licorice combinations. Both ginseng and licorice extracts did not produce any substantial accumulation of sub G1 cells at the LC50 concentration tested. With the exception of GE at 24 h, analysis of the G1 phase of the cell cycle indicated that there was a significant (*P* < .05) cell-cycle arrest for all treated cells as compared to the control at 24 to 72 h. In addition, a significant (*P* < .05) reduction in cell percentages at the G2/M phase was seen at 72 h. G1 cell-cycle arrest data are in agreement with the reported literature, licorice extract have been reported to inhibit cell proliferation, and induce G1 phase arrest in an estrogen sensitive breast cancer cell line (MCF-7) [28] and affect viability in cultured prostate cancer cells (LNCaP) [11].

One possible explanation of these antagonistic effects on cell viability is that the active compounds in ginseng and licorice extracts may compete for the same cellular receptor. In the ginseng extract, ginsenoside Re was most abundant saponin at 20.2% dry weight, followed by ginsenoside Rb1 (8.8%), Rg1 (1.5%) and Rd (0.7%). Ginsenosides such as Rg1, a protopanaxatriol type dammarane saponin that includes structurally related ginsenoside Re, has been reported to be a functional ligand of the glucocorticoid receptor [29] and oleanane-type saponins similar to glycyrrhizic acid determined to be 7.1% in the extract have been compared to dexamethasone, a synthetic glucocorticoid in cell culture experiments [30]. An abundance of active compounds would saturate a receptor site leading to lower than expected cellular response than predicted.

Alternatively, licorice under specific conditions can exhibit hepato-protection. For example, glycyrrhizin, structural related to glycyrrhizic acid has been reported to alleviate intraperitoneal induced liver injury by carbon tetrachloride (CCl_4_) in mice by down regulation of pro-inflammatory markers [31]. Pre-treatment of cultured hepatocytes with licorice or glycyrrhizic acid protected against azathioprine injury through increasing the intracellular glutathione levels. Nakamura et al. reported that glycyrrhizin caused a dose-dependent inhibition of LDH leakage caused by CCl_4_ in primary rat hepatocytes [32]. This protective effect was evident in this study as fractions that contain more licorice than ginseng reduced the cytotoxic effect and membrane permeation of ginseng. Licorice may protect against ginseng cytotoxicity and thus leading to the observed antagonism and increase in hepatocyte survival.

Combinations of ginseng to licorice extract using a fix fraction isobolographic analysis were found to be antagonistic rather then synergistic and reduced the cytotoxicity compared to the individual extracts. Furthermore, licorice extract was found to reduce ginseng extract membrane permeation properties. Further research is needed to determine the precise cellular trigger and if these observed effects are cell specific.

## Funding

Ministry of Education, Singapore; National University of Singapore (NUS) (Grant R-143-00-340-112).

## Figures and Tables

**Table 1:** Theoretical and observed LC50 values for ginseng and licorice extracts.

Fractions^a,b^	Theoretical^b^ LC50 CAL50 (mg/mL)	Observed LC50^c^ EXPLC50 (mg/mL)	Difference (%)
f1/5	0.56	0.65 ± 0.01^d^	16.0
f1/3	0.57	0.70 ± 0.02^b,c^	22.9
f1/2	0.59	0.75 ± 0.02^a,b^	21.3
f2/3	0.60	0.74 ± 0.02^a^	18.1
f4/5	0.62	0.69 ± 0.02^c^	11.2

^a^For example, f1/5 refers to the amount of ginseng (1/5) to licorice (4/5) in the mixture.

^b^Refer to the Methods section for a description of the calculations and variables CAL50 and EXPLC50.

^c^Values with different letters are significantly different (*P* < .05).

**Table 2:** Cell-cycle analysis.

Cell cycle	Sub G1		G1		S		G2/M	
Time (h) 24								
Control	0.46 ± 0.27	a	63.19 ± 0.83	a	14.10 ± 0.51	b	20.08 ± 0.83	a
GE	2.05 ± 2.21	a	68.18 ± 5.30	ab	9.39 ± 2.52	ab	19.61 ± 3.39	a
LE	0.79 ± 0.26	a	73.4 ± 2.50	bc	9.54 ± 1.55	ab	15.52 ± 1.76	a
f1/5	0.46 ± 0.05	a	75.01 ± 1.50	bc	9.74 ± 1.87	ab	14.07 ± 2.08	a
f1/3	0.26 ± 0.08	a	76.21 ± 2.01	bc	8.04 ± 1.92	a	14.63 ± 2.50	a
f1/2	0.25 ± 0.03	a	77.34 ± 2.59	c	8.18 ± 2.65	a	13.93 ± 3.01	a
f2/3	0.27 ± 0.01	a	76.86 ± 3.55	bc	8.54 ± 0.47	a	13.76 ± 2.85	a
f4/5	0.31 ± 0.06	a	77.37 ± 4.24	c	7.75 ± 2.42	a	14.12 ± 3.73	a
Time (h) 48								
Control	0.40 ± 0.36	a	69.61 ± 0.85	a	10.54 ± 3.09	a	17.74 ± 3.47	b
GE	0.97 ± 0.21	b	77.31 ± 1.76	b	6.86 ± 2.25	a	14.17 ± 1.47	ab
LE	0.67 ± 0.11	ab	78.47 ± 0.65	bc	8.88 ± 1.47	a	11.79 ± 1.63	ab
f1/5	0.32 ± 0.08	a	80.38 ± 0.73	bcd	6.72 ± 2.22	a	12.29 ± 2.86	ab
f1/3	0.31 ± 0.02	a	82.48 ± 1.56	cd	6.27 ± 2.53	a	10.8 ± 1.63	a
f1/2	0.26 ± 0.16	a	82.92 ± 1.76	d	5.76 ± 2.06	a	10.84 ± 2.89	a
f2/3	0.29 ± 0.13	a	83.17 ± 1.77	d	5.08 ± 1.21	a	11.20 ± 0.96	a
f4/5	0.35 ± 0.14	a	82.39 ± 2.11	cd	5.36 ± 1.31	a	11.45 ± 1.19	a
Time (h) 72								
Control	0.54 ± 0.12	a	65.76 ± 2.02	a	10.38 ± 4.60	a	20.17 ± 2.14	c
GE	1.14 ± 0.27	c	75.73 ± 0.61	c	6.10 ± 2.87	a	16.15 ± 2.83	bc
LE	1.04 ± 0.11	bc	79.44 ± 0.62	bc	8.71 ± 0.70	a	10.90 ± 0.63	ab
f1/5	0.69 ± 0.23	abc	80.68 ± 0.24	abc	6.80 ± 3.04	a	11.91 ± 2.55	ab
f1/3	0.73 ± 0.16	abc	81.68 ± 0.62	abc	5.95 ± 2.47	a	11.60 ± 2.36	ab
f1/2	0.63 ± 0.02	ab	83.27 ± 1.07	ab	4.77 ± 2.04	a	11.16 ± 1.44	ab
f2/3	0.45 ± 0.12	a	82.93 ± 0.22	a	6.55 ± 2.69	a	9.87 ± 2.12	ab
f4/5	0.42 ± 0.19	a	79.31 ± 1.50	a	7.98 ± 2.74	a	11.82 ± 1.39	ab

Columns of the same category and time period with different letters are significantly different (*P* < .05).
